# Invasive candidiasis and oral manifestations in premature newborns

**DOI:** 10.1590/S1679-45082013000100013

**Published:** 2013

**Authors:** José Endrigo Tinoco-Araujo, Diana Ferreira Gadelha Araújo, Patrícia Gomes Barbosa, Paulo Sérgio da Silva Santos, Ana Myriam Costa de Medeiros

**Affiliations:** 1 Universidade de São Paulo, Bauru, SP, Brazil.; 2 Universidade Federal do Rio Grande do Norte, Natal, RN, Brazil.

**Keywords:** Candidiasis, invasive/epidemiology, Candidiasis, oral/epidemiology, Candidiasis, oral/diagnosis, Infant, premature

## Abstract

**Objective:**

To investigate prevalence of invasive candidiasis in a Neonatal Intensive Care Unit and to evaluate oral diseases and *Candida spp*. colonization in low birth weight preterm newborns.

**Methods:**

A descriptive epidemiological study performed in two stages. First, prevalence of candidiasis was analyzed in a database of 295 preterm patients admitted to hospital for over 10 days and birth weight less than 2,000g. In the second stage, oral changes and *Candida spp*. colonization were assessed in 65 patients weighing less than 2,000g, up to 4 week-old, hospitalized for over 10 days and presenting oral abnormalities compatible with fungal lesions. Swab samples were collected in the mouth to identify fungi.

**Results:**

Prevalence of candidiasis was 5.4% in the database analyzed. It correlated with prolonged hospital length of stay (p<0.001), in average, 31 days, and 85% risk of developing infection in the first 25 days. It correlated with low birth weight (p<0.001), with mean of 1,140g. The most frequent alterations were white soft plaques, detachable, in oral mucosa and tongue. Intense oral colonization by *Candida spp* was observed (80%).

**Conclusions:**

The frequency of invasive candidiasis was low and correlated with low birth weight and prolonged hospital stay. The most common oral changes were white plaques compatible with pseudomembranous candidiasis and colonization by *Candida spp*. was above average.

## RESUMO

**Objetivo:**

Investigar a prevalência de candidíase invasiva em Unidade de Terapia Intensiva Neonatal e avaliar alterações bucais e colonização bucal por *Candida spp*. em recém-nascidos prematuros com baixo peso.

**Métodos:**

Estudo epidemiológico descritivo em duas etapas. Na primeira, analisou-se prevalência de candidíase invasiva em base de dados de 295 prematuros com tempo de internação superior a 10 dias e peso ao nascer inferior a 2.000g. Na segunda etapa, avaliaram-se alterações bucais e colonização por *Candida spp*. em 65 pacientes com peso inferior a 2.000g, com até 4 semanas de idade, internados há mais de 10 dias e apresentando alterações bucais compatíveis com lesões fúngicas. Coletaram-se amostras com *swab* bucal e identificou-se a colonização fúngica.

**Resultados:**

Na análise da base de dados, constatou-se que a prevalência de candidíase foi de 5,4%. Houve correlação com o tempo prolongado de internação (p<0,001), que foi, em média, de 31 dias, com risco de desenvolver infecção de 85% nos primeiros 25 dias. Houve correlação com o baixo peso ao nascer (p<0,001), com média de 1.410g. No exame dos pacientes, constatou-se que as alterações bucais mais frequentes foram placas brancas, moles, destacáveis localizadas na mucosa jugal e língua. Houve colonização bucal expressiva por *Candida spp*. (80%).

**Conclusão:**

A frequência de candidíase invasiva foi baixa e teve correlação com baixo peso ao nascer e tempo de internação prolongado. As alterações bucais mais frequentes foram placas brancas compatíveis com candidíase pseudomembranosa e a colonização por *Candida spp*. foi acima da média.

## INTRODUCTION

Invasive candidiasis is a high impact disease in neonates. It affects 2 to 20% of preterm newborns (NB) representing 10% of all sepsis cases in low-weight neonates (<1,500g) and the second cause of mortality due to opportunistic infections in very low-weight preterm NB (<1,000g)^(1–4)^. Diagnosis is made by fungal culture in blood, urine, and cerebrospinal fluid (CSF)^(2)^. Mortality due to invasive candidiasis is high in preterm NB, ranging from 20 to 50%^(4–8)^. The condition can be endogenous, starting with the colonization of the gastrointestinal mucosa^(9)^. The mouth is an entry for opportunistic fungal infections and oral candidiasis is a possible primary source of gastrointestinal colonization and systemic dissemination of the fungal infection through the mesenteric capillaries. Thus, oral manifestations of candidiasis in preterm NB and the oral care to prevent and control new cases in which the mouth was the entry site should be well understood.

Perinatal transmission of *Candida spp*. can be vertical, transmitted from mother to baby during birth, or horizontal, due to external contamination^(7,8)^. Other relevant risk factors are: prematurity and immaturity of the immune system, weakened physical barriers, colonization, and exposure to environmental factors, such as central venous catheter, prolonged use of endotracheal tube, H2 blockers, prolonged use of antibiotics or steroids, parenteral nutrition, long hospital stay, recent surgery, dialysis, maternal hypertension, restricted intrauterine growth, asphyxia, hospital infection and advanced maternal age at gestation^(2,3,5–8,10–15)^.

Candidiasis is the most frequent opportunistic infection in preterm NB and it has different presentations in immunocompromised status, therefore sometimes being difficult to diagnose. Mucocutaneous pseudomembranous candidiasis or yeast infection - popularly known as thrush, is the most common form and is characterized by white plaques on the oral mucosa similar to sour milk. These plaques are made of hyphae, epithelial cells, and necrotic tissue and can be removed. The underlying mucosa can be normal or erythematous. In the erythematous type there are red patches instead of white plaques. In the chronic hyperplastic form there are white non-detachable plaques; it is also known as *Candida spp*. leucoplakia^(16–18)^.

Fungi are resident microbiota colonizing 10 to 60% of preterm NBs^(19)^. About 50% of these patients are colonized by *Candida spp.* during the first week of hospitalization. Candida yeasts are the most common ones, according to several studies on fungal infections and the most frequent species is *Candida albicans* that accounts for 75% of the opportunistic fungal infections, followed by *Candida glabrata*, *Candida krusei*, *Candida tropicalis* e *Candida parapsilosis* ^(6–8,20–22)^. As skin and mucosa colonization is decisive for the development of invasive candidiasis^(23,24)^, it is believed that oral hygiene eliminating any residues that might be used as culture medium is important to minimize *Candida spp*. Colonization and prevent the dissemination of these microorganisms during hospital stay.

## OBJECTIVE

To investigate the prevalence of invasive candidiasis in a Neonatal Intensive Care Unit (NICU) and to assess oral manifestations and colonization by *Candida spp.* in preterm NB at this unit.

## METHODS

This study was approved by the Committee of Research Ethics on Human Beings of the Universidade Federal do Rio Grande do Norte (CAAE number 0002.1.051.000-08). No medical interventions were performed.

This is a descriptive epidemiological study conducted in two stages. First, a prevalence study was performed using a database to understand the distribution of candidiasis at the NICU. Records of patients admitted during a two-year period were analyzed. A total of 295 patients meeting inclusion criteria of hospital length of stay >10 days, birth weight <2,000g were included in this study. Data collected included diagnosis of candidiasis, length of hospital stay, gender, type of delivery, birth weight, gestational age and maternal age. The records contained no information on oral manifestations.

Second, the specific aim of this study – the study of oral manifestations of *Candida spp*. colonization – was assessed. However, during this stage patients were examined and material was collected with a swab for laboratory analysis, thus establishing a case series. A total of 65 newborns weighing ≤2,000g, up to fourweek old, hospitalized for over 10 days, presenting oral manifestations compatible with fungal lesions and not receiving medical treatment for fungal infection were included. An assessment protocol with medical and demographic data was completed for each patient. Parents or gurdians signed the informed consent.

Whenever lesions probably due to *Candida spp.* characterized by soft, white, and detachable plaques on erythematous surface were detected, samples were collected rubbing a sterile swab on the oral mucosa. This material was spread in tubes containing Sabouraud dextrose agar medium. Isolates generating yeast-like colonies were submitted to the germination test in order to identify *C. albicans* or *C. dubliniensis.* A small amount of yeast was inoculated in tubes containing 0.5ml of saline solution to be tested and compared to positive and negative controls of standard strains of *C. albicans* or *C. glabrata,* respectively*.* These tubes were incubated at 37°C for 2 hours and then a drop was deposited on slides covered by cover slips and examined with optic microscope.

### Statistical analysis

The data obtained were tabulated and submitted to statistical analysis using the Statistical Package for the Social Science (SPSS), version 15.0, and results were presented in absolute and relative frequencies; correlation tests used a 95% confidence interval (95% CI).

## RESULTS

Using medical records, the prevalence studies analyzed data of 151 (51.2%) boys and 144 (48.8%) girls totalizing 295 patients with no significant differences regarding gender. Candidiasis was present in 16 (5.4%) patients.

Table 1 describes the variables “length of stay”, “birth weight”, “gestational age” and “maternal age”. Table 2 shows correlation between candidiasis and prolonged stay (mean=31 days); the probability of candidiasis is higher during the first 25 days of hospitalization (p<0.001) with an 85% risk and 95%CI (0.95, α=0.05). Table 2 also illustrates the correlation between birth weight that varied between 530 and 2,000g (mean of 1,140g). There were 169 (57.2%) low weight (<1,500g) preterm NB who had higher risk of infection and of these 8.2% (n=14) had candidiasis (p<0.001). There was no correlation between gestational age and maternal age.

**Table 1:** Characterization of the sample using length of stay, birth weight, gestational age and maternal age

Variables	Minimum	Maximum	Mean	SD
Length of stay (days)	11	130	31.33	20.29
Birth weight (g)	530	2,000	1,410	320
Gestational age (weeks)	22	40	32.84	2.39
Maternal age (years)	13	44	24.55	7.09

SD: standard deviation

**Table 2:** Correlation between candidiasis, length of stay and birth weight

Characteristics	Length of stay			
	<25 days	>25 days	Total	p value
Healthy	153	126	279	0.001
Candidiasis	2	14	16	0.001
Total	155	140	295	0.001
**Characteristics**	**Birth weight**			
	**<1,500g**	**>1,500g**	**Total**	**p value**
Healthy	155	124	279	0.001
Candidiasis	14	2	16	0.001
Total	169	126	295	0.001

After assessing the medical records and detecting the prevalence of candidiasis in preterm NB, 65 patients with oral manifestations with pseudomembranous Candida-like lesions were examined. They presented white, soft, detachable lesions on the tongue and oral mucosa. The microscopic examination showed colonization in 52 patients (80%), in that, *C. albicans* was found in 46 (70.7%), *C. glabrata* in 4 (6.1%) and *C. krusei* in 2 (3%) patients.

There was no significant correlation between fungal colonization and the variables “length of stay”, “birth weight”, “gestational age” and “maternal age”, although higher colonization rates were found in mothers aged >23 years and gestational age >30 weeks.

## DISCUSSION

Invasive candidiasis is the second cause of mortality due to infectious diseases in preterm NB and represents 10% of all sepsis cases. It is the major cause of late infection in preterm NB^(2,3,5,12)^ with varying incidence and high mortality^(4–8)^. Long hospital stay and exposure to environmental factors, as well as contact with other patients or transmission by health professionals with hands colonized with fungi are important causes of opportunistic infections^(25)^. The prolonged use of broadspectrum antibiotics is another relevant risk factor that can lead to colonization of the gastrointestinal tract and trigger systemic infection^(26)^.

In this prevalence study based on NICU records, a correlation between candidiasis cases and low birth weight^(4)^ as well as with long hospital stay was observed. The greatest risk of infection occurs between the second and fourth weeks of life. The longer the hospital stay, the greater the use of central venous catheter, medications, and exposure to environmental risk factors^(10,11)^. Oral colonization by *C. albicans* was found in 70% of the cases and the most frequent oral manifestations were pseudomembranes formed by fungi, cheratotic residues, inflammatory and epithelial cells, bacteria and fibrin^(27)^.

The prevalence of candidiasis observed when analysing 295 records is corroborated by the literature. This result may be related to antifungal prophylactic treatment and to appropriate use of biosafety measures as a result of investment in human resources and continued education of physicians and nurses. Even when such care is taken, the immunocompromised status of these patients is a risk factor for *Candida spp.* dissemination. Low birth weight indicates immune system imaturity and fragile physical barriers and, although neutrophil counts in preterm NB are similar to those in adults, neutrophil function and mobilization in preterm NB are different when facing infections; neutropenia occurs due to small neutrophile bone marrow reserves that are quickly depleted^(13)^.

The diagnosis of oral candidiasis is based on medical signs and symptoms and therefore the participation of a dental surgeon is important to assess patients suspected to have an opportunistic fungal infection. Some additional tests, such as exfoliative cytology with direct search for fungi and microbiologic culture can be used, since both have few false-positive results^(17)^. Early diagnosis of oral candidiasis in preterm NB allows immediate topical treatment with hygiene, elimination of pseudomembranes and topic use of antifungal medication, thus preventing invasive candidiasis that increases morbitity in the NICU.

In this case series oral colonization by *Candida spp*. was found in most patients examined whose collected samples were sent to microscopic analysis. This figure indicates high colonization when compared to other papers reporting *Candida spp.* oral colonization rates of 26.7^(28,29)^, 39.4, and 70%^(9)^. However, when analyzing this NICU medical records, the low frequency (5.4%) of invasive candidiasis suggests that prevention and treatment carried out by the multidisciplinary team have been effective to control hematogenous dissemination and reduce infections.

According to these findings, the use of an oral care protocol for these patients is suggested in order to reduce colonization of the oral biofilm by pathogens and prevent systemic complications^(30)^. This protocol stresses the importance of individual protection equipment after careful handwashing, raising the patient' head to reduce the risk of aspiration of oral secretions and, whenever needed, aspiration, good hygiene of the mouth with swabs moistened with antimicrobial solution to reduce the risk of opportunistic infections. The best options are 0.12% aqueous solution of chlorhexidine digluconate, lactoperoxidase enzyme solution^(31)^ or 1.5% hydrogen peroxide solution. Patients' lips should be kept moistened with pure lanoline cream^(32)^ to minimize fissures that allow the entry of microorganisms^(30)^.

## CONCLUSIONS

The frequency of invasive candidiasis was low and the most important triggers were low birth weight and prolonged hospital stay with an 85% risk during the first 25 days at hospital.

*Candida spp*. colonization was found in 80% of patients and the most frequent oral manifestations were white, soft plaques resembling pseudomembranous candidiasis that can potentially lead to invasive candidiasis.
